# Modeling a Pandemic (COVID-19) Management Strategy for Urban Slums Using Social Geometry Framework

**DOI:** 10.1057/s41287-020-00317-5

**Published:** 2020-10-26

**Authors:** Francis Onditi, Moses Obimbo, Samson Kinyanjui Muchina, Israel Nyadera

**Affiliations:** 1 School of International Relations and Diplomacy, Riara University, P.O.BOX, Nairobi, 49940-00100 Kenya; 2 School of Medicine, University of Nairobi, P.O.BOX, Nairobi, 30197-00100 Kenya; 3 Kenya Medical Research Institute (KEMRI)/Wellcome Trust Research Programme, Nairobi, Kenya; 4 Department of Political Science, Macau University, Taipa, Macau China

**Keywords:** Social geometry, Containment strategy, COVID-19, PHI, Informal settlement, Kenya

## Abstract

The purpose of this paper is to utilize social geometry framework to model a pandemic (COVID-19) management strategy in densely populated informal settlements in Kenya. Our central claim is that the containment strategy that was instituted to control spread of COVID-19 failed to recognize the socio-cultural and livelihood complexities of the urban slum residents. This unmitigated strategy predisposed the residents to risks of heightened transmission of the pandemic. Drawing on social geometry approach in the analysis of human relations, we reveal some insights offered by our experiences in theorizing about public health intervention (PHI) and in doing so develop an alternative analytical framework (‘social pendulum’) to support the development of a PHI strategy that is compatible with the *swing-like* lifestyle of residents in the informal settlements. Our conclusion revisits the reliability and validity criteria for the new framework and offers some direction for further research.

## Resumé

Cet article a pour but d'utiliser un cadre de géométrie sociale pour modéliser une stratégie de gestion de la pandémie (COVID-19) dans les zones d’habitation informelles et densément peuplées au Kenya. Nous partons du principe que la stratégie d'endiguement qui a été mise en place pour contrôler la propagation de la COVID-19 n'a pas réussi à prendre la mesure de la complexité socioculturelle, ni celle des moyens de subsistance, des habitants des bidonvilles urbains. Cette stratégie indiscriminée a exposé les habitants à des risques accrus de transmission de la pandémie. Le modèle de géométrie sociale, au contraire, a la possibilité d'influencer la refonte d'une stratégie alternative d'intervention de santé publique qui serait compatible avec la configuration unique des moyens de subsistance dans les zones d’habitation informelles, configuration qui adopte des mouvements de pendule. Nous élaborons un cadre analytique (du «pendule social») en nous basant sur ce mode de vie, pour appuyer le développement d'une stratégie alternative d'intervention de santé publique. Dans notre conclusion, nous revisitons les critères de fiabilité et de validité du nouveau cadre et offrons une orientation pour les études à venir.

## Introduction

Containment strategies to control the transmission of COVID-19 were adopted around the world. However, few studies have considered what ‘containment’ means for the people living in high-density informal settlements such as those in Kenya. Kenya’s containment strategy was enforced through a number of restrictive measures: curfew (dusk to dawn), workplace closure, isolation, cancelation of mass gatherings, workplace distancing, and school closure. As a result, ‘containment’ was presented as a ‘new normal.’ However, the challenge with the ‘new normal’ strategy of ‘containment’ is that it obfuscates and denies the inequalities of ‘normal.’ From a political economy point of view, the so-called ‘new normal’ also includes policies that have further marginalized people living in slums. This structural disruption in urban settlement has wide and deep implications on the social geometries of life. Black (1976), defines social geometry as the social structure of behavior among individuals or a collective. For instance, families in slum areas rely on their social networks to survive through their livelihood needs and to seek social protection through collective actions. If this lifestyle is suddenly disrupted by restrictive policies, this community would find it difficult to survive as they do not have a regular source of income. Moreover, restricting out-movement of residents in such a densely populated area leads to indoor overcrowding, an environmental factor classified by epidemiological researchers as deadly for the spread of respiratory infectious diseases in sub-Saharan Africa (Boyce et al. 2019). This is more so because the coronaviruses can lead to severe lower respiratory tract infections and acute respiratory distress syndrome (ARDS).

In light of these risks, it is reasonable to observe that the ‘containment’ strategy could potentially disrupt the individual’s and group’s lifestyle. Disrupting people’s livelihood increases the likelihood that they become vulnerable to both communicable diseases and socio-economic shocks. Invariably, containment, as a public health intervention (PHI) strategy lacks diversity, promotes massification and constrains access to the already overburdened sanitary facilities. Although WHO (World Health Organization) (cited in Lewnard and Lo 2020), applauded containment as being scientifically robust with significant reduction in transmission of COVID-19 in countries such as China, the socio-economic injustices perpetuated through this strategy might be overstraining to vulnerable populations. In other words, the effectiveness and impact of social control will depend on the efficacy of the intervention strategy and the pre-existing politico-economic conditions of the slum residents (Prem et al. 2020). To this end, researchers have recommended evidence-based interventions as the only way public policy makers can ensure publics’ trust (Lewnard and Lo 2020). Reconciling the different livelihood needs of the slum dwellers and strategies for containment is thus crucial to any meaningful effort towards the management of the pandemic.

We argue that social geometry is a distinctive notion, which goes well beyond containment and other social control models. Given its foundation in human relations, the model could provide important insights for promoting an effective PHI strategy for addressing the various socio-economic and spatial inequalities in the management of pandemics. This strategy aligns well with the epidemiological characteristics of the novel viral contagion, which often is asymptomatic at the time of transmission leading to difficulty in its initial detection and propensity to posing a challenge to regular surveillance in areas with weak health care systems. To develop a balanced PHI strategy that embodies dimensions of social geometry remains a challenge for public health actors and scholars concerned with the promotion of equal access to health care. Thus, the intersection between the *social-theoretical* foundations of social geometry and the vulnerabilities of slum dwellers to a pandemic form the core of this paper.

In this paper, the introduction is proceeded with the methodological considerations. The next section provides an overview of the unfolding epidemiological situation and the various approaches deployed to control the spread of the coronavirus in Kenya. In the next section, we theorize public health intervention (PHI) by comparing human interaction to social geometry of life. Based on the spatial vulnerabilities in slum areas, we inductively construct (modeling) various decision scenarios using the pairwise ranking technique. Finally, we identify elements within the typologies of social geometry that would constitute an effective analytical framework for informing a PHI strategy in the management of future pandemics. We conclude that strategies for managing pandemic must be inclusive, innovative, and based on the socio-geometric realism of people living in the informal settlements.

## Methodological Considerations

**Fig. 1:**
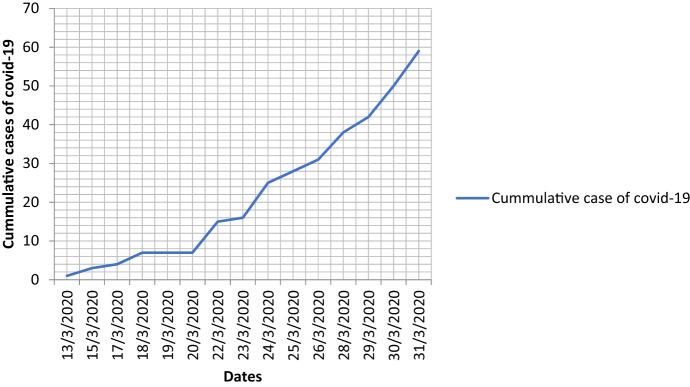
Daily cases of COVID-19 transmissions before enforcing the containment strategy. *Source* Authors’ Compilation based on the data obtained from the Republic of Kenya’s Ministry of Health; Daily transmission of COVID-19 across the 47 counties. Analysis and plotting of the graph are based on infection cases tabulated in Annex 1

**Fig. 2:**
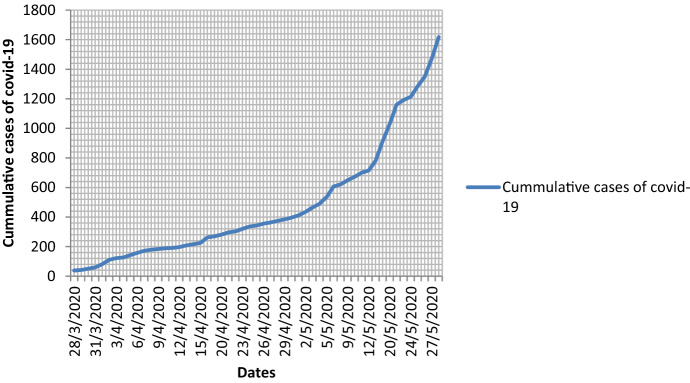
Daily cases of COVID-19 infection after enforcing the containment strategy. *Source* Authors’ Compilation based on the data obtained from the Republic of Kenya’s Ministry of Health; Daily transmission of COVID-19 across the 47 counties. Analysis and plotting of the graph are based on infection cases tabulated in Annex 1

This study combines methods including content analysis, documentary review of the social geometry (SG) framework, social modeling (scenario building) as well as validation of the SG to the existing vulnerability qualitative data of the urban populations living in slums. The study involved three steps. The first step was to ascertain if the ‘containment’ strategy reinforced the theoretical assumptions regarding the control of the community transmission of COVID-19 as claimed in the literature. One of the predominant assumptions on the usefulness of containment as a transmission control strategy was based on the understanding that restricting movements would minimize human to human contacts, hence reduce community transmission. Using the *Google Mobility Reports* from March 28, 2020 to May 27, 2020, we were able to establish the relationship between rates of transmission and the enforcement of the containment strategy (See Figs. 1, 2). Use of the Republic of Kenya’s Ministry of Health daily transmission of COVID-19 guaranteed the authenticity of the data.

In the second step of the study, we conducted systematic review of the extant literature grounded in the Donald Black’s (1976) social geometry approach to analyses of human relations. More than 20 articles published by Black and/or other scholars on social geometry over three decades (1976–2013) were analyzed. The periodization (1976–2013), was arrived at based on the evolution of the concept of social geometry. While 1976 marks the beginning of theorization of the concept, 2013 captures the period when most articles published on this notion were applied in the analysis of human relations. These articles were available online and we had access through the paywall and not duplicates. We considered all literature items including books, book chapters, journal articles, and conference papers. Articles were coded as per the *visibility* of the three building blocks of the SG (direction, distance, and location) (Black 1976; 1998; 2000). These three elements are central to both spatial and social distances in the study of human relations. Our visibility coding was as follows: (1⇒high) application of SG to social relations, (2⇒medium) besides identification, the three elements were mapped and analyzed, and (3⇒low) the three items were identified and defined. These levels of visibility were key in enabling researchers identify four discursive geometric tools (form, style, quantity, and multidimensionality). These tools were later on applied in *reimagining* an alternative PHI strategy. Social scientists have acknowledged this approach to knowledge development as an integral part of discursive ontologies (Brown 2017).

The final step utilized Pairwise Ranking technique, commonly known as Perron Rank. This technique was preferred because it supports our qualitative modeling (scenario development) in search of the most suitable strategy for PHI in the informal settlements. In this technique, 16 items considered by the IDSUE (Indicator Development for the Surveillance of Urban Emergencies) data base as ‘risk factors’ were identified from the literature on Kenyan slum conditions (USAID/Concern World Wide 2014). The use of the IDSUE set of indicators was informed by the fact that it is the most preferred approach for responding to slow-onset crises in urban areas. A combination of IDSUE and Pairwise Ranking techniques have been hailed by social scientists in developing arbitrarily different rank order and prioritization of projects, issues affecting the community or policy choices (Tran 2013). The method was ideal for this study as it assisted researchers to theoretically model various scenarios by comparing the three possible strategic options (containment, lockdown, and social pendulum). To achieve this comparison, we used codes to assign the risk levels of the three strategies against each one of the 16 ‘risk factors’ as follows: Red⇒high risk, Yellow⇒medium risk, and Green⇒low risk.

The above set of data and methods are useful in understanding how the socio-economic and political determinants of health influence the susceptibility of slum residents to risks of a pandemic. The following section sets the stage by evaluating the general assumptions on the role of containment strategy and other social control strategies in preventing the transmission of COVID-19 in Kenya.

## Situational Analysis and Gaps in the Approaches

On 7th January 2020, the World Health Organization (WHO) announced the epidemic disease caused by a novel coronavirus identified from the throat swab of a patient by the Chinese Center for Disease Control and Prevention (CDC). This virus was named SARS-CoV-2 due to severe acute respiratory syndrome it caused. The disease caused by this virus was named coronavirus disease 2019 (COVID-19). This disease was later declared a public health emergency of international concern (PHEIC) on 30th January, 2020 by the WHO (WHO 2020). This is the 3rd of the outbreaks caused by the viruses from the *Coronaviridae* family, the foregoing two having been the severe acute respiratory syndrome coronavirus (SARS-CoV) in 2002, Guangdong, China, and the Middle East respiratory syndrome coronavirus (MERS-CoV), detected in Saudi Arabia in 2012 (Mohd et al. 2016). The Coronaviruses are large, enveloped, single-stranded ribonucleic acid (RNA) viruses infecting mammals and birds. These viruses can lead to severe lower respiratory tract infections and acute respiratory distress syndrome (ARDS).

As of 21st September, 2020, SARS-CoV-2 had been responsible for 30.6 million confirmed cases and 950,000 (3.1%) deaths around the world, 37,489 confirmed cases and 669 (1.9%) deaths in Kenya alone (European Centre for Disease Prevention and Control 2020). These cases and deaths occurred as a result of rapid local transmissions through local travels, gatherings, and human behavior. Although there exist no antiviral treatment or vaccine for the disease at the time of writing this paper, there are several behavioral methods of preventing and combating the disease; cleaning of hands with alcohol-based sanitizers, avoiding touching one’s face, practicing respiratory hygiene, self-isolation, quarantine, restricting travel, and social distancing (Nyabadza et al. 2020).

Contrary to the speculations which posit that containment strategy would eliminate transmission, the cases in Kenya continued to rise (see Fig. 1 derived from Annex 1), albeit less, compared to the west and other African countries such as South Africa (JP Morgan cited in Mail online 2020). The fact that the transmission of the disease continued and the COVID-19 was not eliminated deserves a reflection. This reflection illuminates the social geometry as a theoretical framework potential of predicting the behavior of slum residents under the strategy of ‘containment.’ In this study, we define ‘containment’ ‘as the non-pharmaceutical measures that were put in place by the Kenyan government to control transmission of COVID-19, including restricted movements and limited social interactions.’ Fig. 1 suggests that during the infant stage of infection characterized by exponential rates of infection, holistic strategies were required to control the factors that led to the increased number of cases, including control of immigration and regulation of internal mobility. However, later on the growth in cases was mainly through community transmission, hence the need to refocus attention to domestic strategies.

In response to the internal community transmission, the Kenyan government introduced various strategies including, ‘containment’ on 27 March, 2020, restricting movement. However, from Fig. 2, it is clear that the infection cases continued to rise, despite the strategy.

The trend in the number of cases of infection in Fig. 2 suggests four noteworthy feedback to the containment strategy that might drive or counter the exponential in transmission of the pandemic in future: (1) there was need for more aggressive and multi-layered approaches targeting counties that were most affected; (2) rapid surveillance and detection of exposed cases and the discovery of asymptomatic cases should have been implemented simultaneously; (3) strengthen the intergovernmental coordination structures and build predictive and early warning capabilities; and (4) shifting the efforts from reactive intervention to preparedness of the most affected counties in the country including robust testing system and monitoring people’s mobility.

The overall mobility trend in Kenya decreased after the containment strategy was instituted on March 26, 2020 (see Annex 2) (Google Report as at April 2020/https://www.google.com/covid19/mobility/). It is evident from Annex 2 that visits to retail and recreation facilities decreased significantly (− 50%), followed by transit stations (− 47%). The report further shows that people visiting grocery stores and pharmacies decreased (− 39%) as soon as the containment strategy was enforced. However, in both cases there were still movements due to essential workers providing essential services. But the opposite trend was observed between workplaces (− 19) and residential spaces (+ 20) (see Annex 2), understandably because during the containment period there were companies and institutions providing essential services still operating and movement within residential spaces was inevitable. It is not clear whether containment had any impact on the prevention of transmission of COVID-19, as seen from Fig. 2 above, the number of new infections continued to increase with spikes being experienced between March 28 and May 25. From the county reports, we can also observe that although Nairobi (− 58%) experienced a significant decrease in mobility, it continued to record spikes in the new cases of infection. A similar trend was established by JP Morgan, who concluded that the virus seems to have had its own dynamics to the extent that the lockdowns did not alter the rate of infections (cited in Mail online 2020). As such, we designed a simulation exercise on the movement of residents in order to establish what the “other” factors driving transmission could have been.

**Fig. 3:**
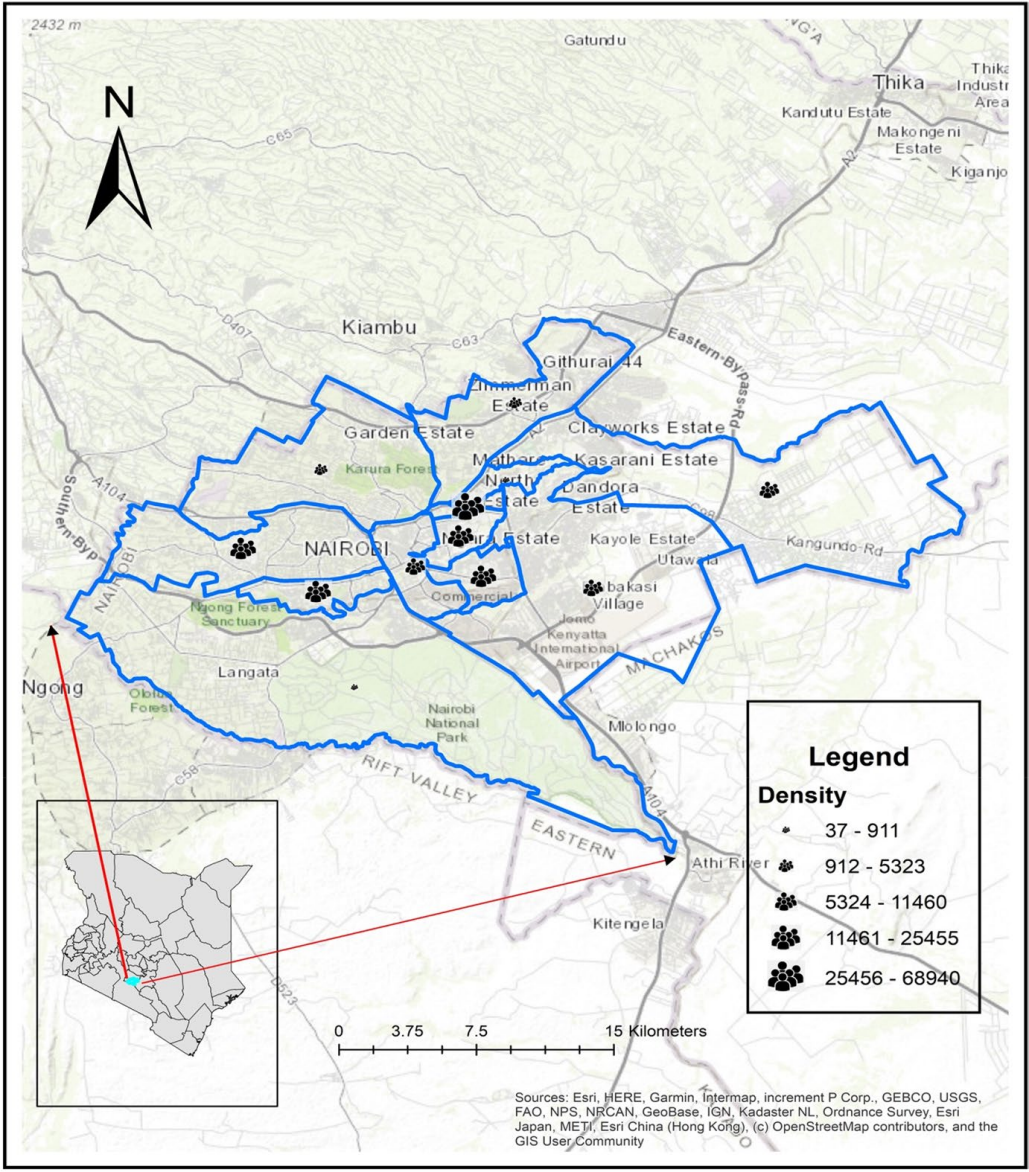
Comparing population density in Nairobi sub-counties. *Source* Authors’ compilation in collaboration with University of Nairobi’s Department of Geography and Environmental Studies

In order to gauge the movement (swing) of people in and outside the *nuclei* of the informal settlement, we simulated the *swing* as illustrated in Annex 3a, b. Annex 3a shows the behavior of slum residents *before* the containment strategy was enforced. As earlier indicated, the livelihood of people in the informal settlement is erratic; the most viable mode of survival is ‘moving away’ from the *community nuclei (*the physical space occupied by slum residents*)*. The erratic movement serves several societal functions; residents move in order to secure food, water, and safety. Also, this irregular movement of the residents is a form of coping mechanism in their efforts to deal with the burden of providing for the family (‘family responsibility escape routes’). To the contrary, when the residents’ movement is restricted, as was the case for the containment strategy, diffusion of people towards the community nuclei (Annex 3b) suddenly increases the pressure on available spatial space and resources. This condition is exacerbated by the unprecedented high population density in the Nairobi slum areas. The population density of Nairobi between 2009 (4515 persons per sq. km) and 2019 (6247 persons per sq. km) increased by 28% (Kenya National Bureau of Statistics 2019). The Nairobi city is also home to the country’s most densely populated sub-counties (see Fig. 3), with the highest being Mathare informal settlement (68,941 persons per sq. km), compared to the middle-class residential area, Langata, with only 911 persons per sq. km (Kenya National Bureau of Statistics 2019).

Unsurprisingly, the urban agglomerations shown in Fig. 3 also happen to be areas experiencing all forms of poverty and irregular micro-migration patterns of livelihood (Kenya National Bureau of Statistics 2019). Owing to this dense population in slums, the community nuclei are extremely overcrowded rendering spatial distancing practically difficult to achieve. This skewed population distribution in Nairobi has implications on mobility of the residents.

**Fig. 4:**
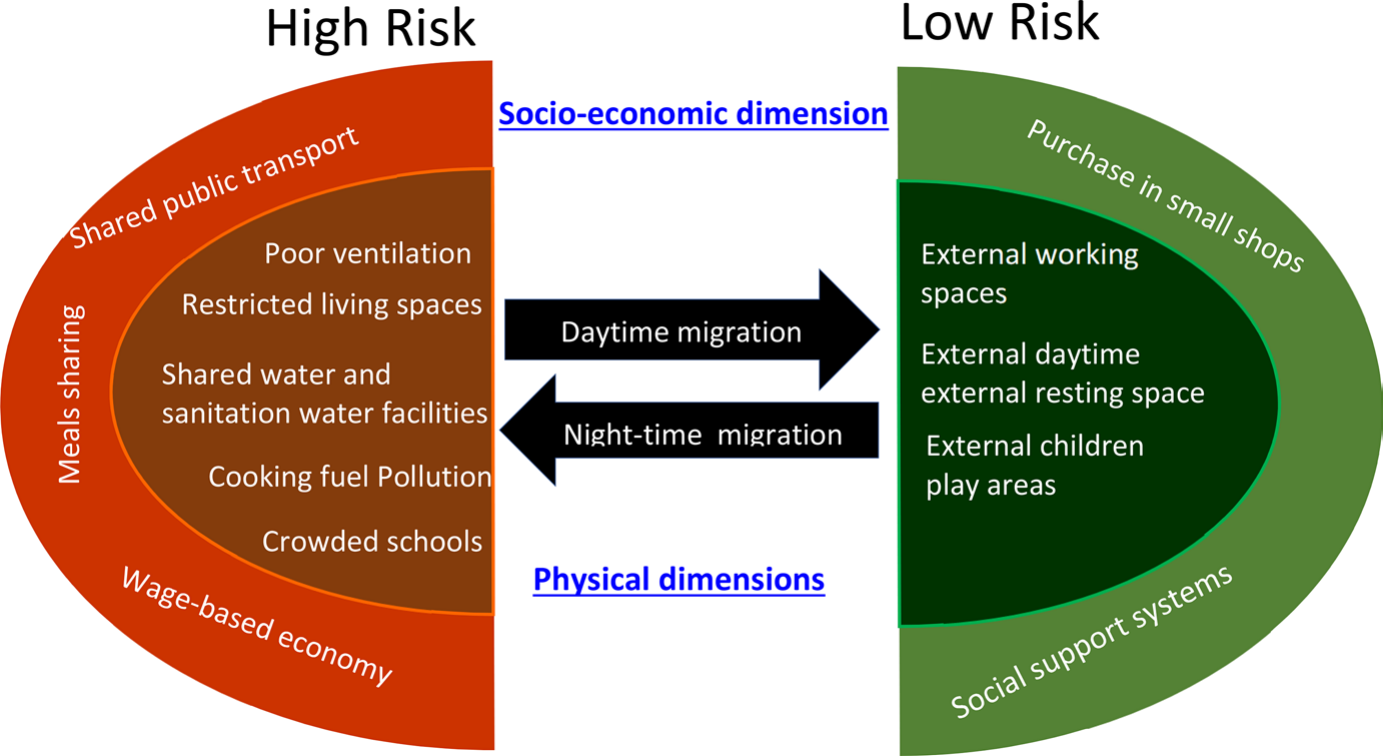
Results of simulation when slum residents responded to the introduction of containment strategy. *Source* Authors’ compilation by simulating the micro-migratory behavior of slum residents and changes in the profile of the “risk factors,” as a derivative of Annex 3a, b

From Fig. 4 (a derivative of Annex 3a, b), it is plausible to observe that the changing pattern of movement of people (day and night) across the continuum as was triggered by the containment strategy would eventually increase the risks of slum residents to a heightened rate of transmission. Within the same ecosystem (see the dark green shadings of Fig. 4), there are pockets of safe spaces available for working, resting, and playing ground for children. However, the government’s effort to improve on these safe spaces through social systems are often thwarted by neo-patrimonial power politics and a network of vigilante groups controlling access rights in the informal settlements.

In view of the foregoing epidemiological situation and behavioral response from residents, this paper questions the ‘containment’ strategy as instituted by the Kenyan government in preventing the spread of the COVID-19. The strategy assumed that any attempt to prevent further spread of the virus was viable only if it was compatible with the ‘containment’ rules, regardless of the spatial conditions of slum residents. The social distancing, a widespread form of preventing transmission, which here in metaphoric sense implies ‘spatial distancing,’ either endorsed containment or lockdown. Indeed, public health scientists have recently called for ‘spatial distancing’ as opposed to ‘social distancing,’ because the former is grounded in biological and epidemiological stance (Abel and McQueen 2020; Nyabadza et al. 2020). While all these approaches are important for preventing exponential transmission of a pandemic, we subscribe to the view that a PHI framework is best measured through social relations and people’s behavior and the efficacy of that intervention is most appropriately judged by examining resident’s social geometry relative to any containment strategy put in place to manage a pandemic.

## Overview of Social Geometry Analysis Approach

In order to frame empirical evidence appropriately on the current pattern of transmission of COVID-19 and how to improve the PHI strategy, a theoretical framework for explaining the behavior of slum dwellers is necessary. The social geometry model explains how individuals or groups affect and are affected by relationships (vertical and horizontal) (Black 1976). This model has been utilized over the years since its inception in 1976 to guide the data collection and analysis on how social relation phenomena such as cultural closeness and social distance affect population’s perception and reaction to public policies.

In this paper, we apply social geometry on the basis of Black’s (1995) three principles of social relations: (1) in terms of location as social space in relation to others; (2) in a wider purposive sense to refer to social behavior resulting from interactive relationships upward or downward depending on the status of the actors; and (3) as an organizational sense to refer to governmental social control established to achieve a policy goal or strategy in managing public affairs. In the current study, the focus is on the PHI strategy in the management of a pandemic (COVID-19) in densely populated urban areas.

Other concepts from the model which are important for addressing the problem of containment in the management of pandemics are “location,” “direction,” and “distance.” Location in social space can be defined on the basis of five dimensions: stratification, morphology, culture, organization, and social control (Black 1976, pp. 1–2). This implies that every individual in social space function in relation to the others. In the case of managing pandemics (COVID-19), the effectiveness of any PHI will depend on individuals’ relative position and status in the society. For instance, politically “connected” individuals or groups are most likely to access vital services than those considered less “networked” groups such as children and women. Direction and location are interrelated concepts in both social and spatial spaces. But in social space, which is the focus of this paper, human relation interacts in two main directions—upward or downward. Any PHI strategy may trigger directional imbalances giving rise to various forms of inequalities.

**Fig. 5:**
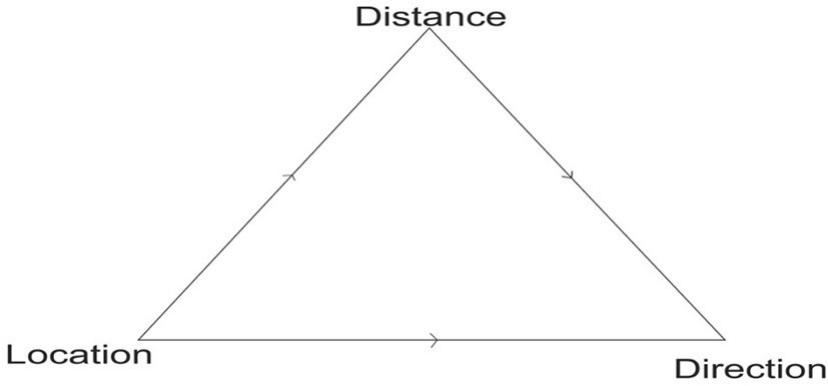
The building blocks of the social geometry of life

In this study, distance is classified as both social and spatial. Without necessarily getting into the controversies of social physics or geometry of law (Black 2002), there is something particularly satisfying about being able to hypothesize, for example, that the implementation of the containment strategy was in fact directly proportional to the rate of transmission of COVID-19 and so on and so forth. That means upward transmissions of the disease varied directly proportional with the distance between two individuals. But, in less individualized societies (such as slums), the term “social distancing” may limit the culture of ‘burden sharing,’ especially in crisis situations. Owing to this contextual and behavioral uniqueness, an effective PHI in the foreseeable future should be evaluated against notions such as *social closeness, migratory behavior, social safety nets*, and *equity*. All these operate within the three building blocks of social geometry (see Fig. 5).

From the foregoing analysis, it is clear that the construct social geometry can play a key role in the design of PHI, therefore its appropriate application should be directly linked to the pattern of lifestyle of slum residents. In this paper, we define social geometry as the, ‘process of designing a PHI that is buttressed on locational human behavior, social class of people, population density, migratory behavior, shared values and social relationships across social spheres of life.’ But management of pandemics can be a complicated process, involving diverse issues and actors beyond the epidemiological characteristics. Therefore, in modeling for an effective pandemic management strategy using social geometry framework, three elements have to be considered: (a) point of intervention among the slum residents; (b) the socio-economic status of the residents and the relationship between the source of the public health policy and the residence will determine whether an intervention succeeds or not; and (c) the case management. In the next section, we build up on these elements as espoused through Donald Black’s (2013) three building blocks of social geometry (location, direction, and distance) to illustrate how a PHI strategy may or may not reconcile with the contextual dynamics.

## Qualitative Modeling and Discussion

In this section, we continue to build the alternative strategy (*social pendulum*) based on socio-geometric constructs developed in the previous sections. The set of constructs are then designated as the *model*, which helps us to develop scenarios of the situation under the ‘old normal’ (without the strategy of containment) and the ‘new normal’ (under the strategy of containment) in the context of a pandemic (COVID-19).

### Why the Containment Strategy Failed on Efficacy

We explore four policy scenarios (*what-if*) that might have impacted on the efficacy of the containment strategy; epidemiological characteristics of the COVID-19, quality of housing, migratory behavior of residents, and social safety nets.

The epidemiological characteristics (transmission and infectivity profile) of COVID-19 remain uncertain. Indeed, SARS-CoV-2 has been found to exhibit high transmissibility potential estimated to be between 2.2 and 3.11 significantly larger than 1. When *R*_0_ is greater than 1, it means that there is a possibility of cases growing exponentially leading to an epidemic or even a pandemic (Zhao et al. 2020; Read et al. 2020). COVID-19 generally carries the course of mild to no clinical symptoms during the incubation period that may last up to 3 weeks, making these people capable of continuing with their daily routines and spreading the infection unperturbed to the unsuspecting population. Other studies have reported that oro-fecal transmission may also be possible (Danchin et al. 2020). Therefore, as projected by our model, suppressive policies such as staying at home and banning non-essential travels can significantly reduce the reproduction rate of the virus. However, although these measures can play crucial role in controlling transmission, the predisposition of slum residents to risk factors such as lack of sanitary facilities and overcrowding is likely to increase their vulnerability to an outbreak or exponential transmissions. This implies that for any PHI to have long-term impact on the population, the strategy must be implemented in tandem with other socio-economic measures. In efforts to address the limitations of mitigation and suppression measures, various predictive mathematical models for epidemics have been proposed. The two most commonly applied include, SIR (Susceptible, Infection, Recovered) and SERS (Susceptible, Infection, Recovered, Susceptible) models, which describe individuals through three mutually exclusive stages of infection (Giordano et al. 2020). It is however, important to note that these models only represent the epidemiological profiling of pandemics, future PHI must stress the need to create a wholistic approach in the management of infectious diseases, including consideration of population density and quality of housing.

The model projection in this paper suggests that outbreak prevention in informal settlements will do relatively little to prevent transmission of the pandemic, provided that 65% of the 4 million people living in Nairobi continue to reside in the informal settlement. This observation has been reinforced by previous studies on why any public policy in informal settlement cannot succeed, unless the issue of population density and quality of housing is adequately addressed (UN-HABITAT 2019). Informal settlements also often have higher levels of intra and intersocial mixing, poor environmental conditions, transient residence, and less regard to human well-beings that makes residents highly vulnerable to infectious diseases (Emina et al. 2011). The implementation of a blanket containment strategy was bound to have an acute negative effect on slum residents who live in *makeshift* single-roomed units made from corrugated iron or mud and often serve as the kitchen, bedroom, and sitting room for a multi-generational family. Our qualitative modeling is consistent with Gibson et al. (2019), who argue that lack of access to quality housing, and regular income has turned the residents into paupers who find themselves in the bustling cities. In short, public accountability has been compromised, thus, as projected by our model, control of community transmission might have not been possible using an intervention mechanism that does not take into account the socio-economic dynamics of the residents.

In regard to household air pollution (HAP), our modeling shows that under business-as-usual (before the strategy of containment), the movement of residents away from the nuclei (as illustrated in Annex 3a), would decongest households while at the same time maintaining social closeness, consequently, reducing the risk of establishing the pandemic through intra-house transmission. However, the trend changes with introduction of containment, as residents are compelled to *swing (move)* inwardly leading to sudden drop in the quality of indoor air. This strategy-induced behavior should be taken as a warning sign that if a PHI is to reach the WHO acceptable quality of air free of indoor pollutants and other hazardous substances, government regulations, and budgetary allocations should be accelerated to improve both indoor and outdoor air conditions. According to our model’s projection without addressing the inequalities and abject poverty, viability of an effective PHI in the foreseeable future is in doubt. Even for the COVID-19, for a downward trend in community transmission to be achieved, especially for asymptomatic cases, poorly ventilated housing structures in informal settlement should have been addressed. Otherwise, the scenario illustrated in Annex 3b will increase the vulnerabilities of residents due to stress on sanitary facilities.

However, if the preventive effect of containment and other social control policies reduces significantly due to civil disobedience by slum residents, the state might establish other alternative measures such as compulsory quarantine and total lockdown, which could become indispensable when the number of infected individuals exceeds the capacity of health care facilities. In the event that suppression of the slum residents by the state fails, and the scenario in Annex 3a remains intact, the lack of alternative means of survival compels the residents to rely on air quality-compromised lighting and cooking facilities. Our model shows that under the baseline scenario (without containment strategy), voluntary self-isolation would be effective. However, the caveat here is that the PHI system should be one that has the capability to detect and mitigate intrahousehold transmission from index cases to contacts.

The continually spiking percentage of transmission of COVID-19 infections in the country, despite instituting the containment strategy, suggests that there are other intervening factors. The effect of school closure, work-from-home, and other mobility restrictions compounded the challenges faced by people living in informal settlements. The assumption by the Kenyan Ministry of Health was that by “containing” people in their homes, they would then redirect investment towards quarantining those infected as an ultimate measure of controlling further transmission. However, asymptomatic cases that accounted for 80% of the infected population, unfortunately turn out to be a significant contributor to the transmission (Ing et al. 2020). The challenge, however, was the identification of such individuals, and especially in informal settlement where residents exhibit irregular migratory behavior. This lifestyle is a unique feature in slums. The residents exhibit *pendulum-like swings* in search of food, job opportunities, new networks, and escaping the scourge of hunger and domestic quarrels. The *swing* is also a sign of personal safety and security. Our model points to the potentially high transmissibility given the irregular micro-migratory behavior of the residents. Factors contributing to this susceptibility are many: the mode of transport is a concern since many residents rely on public means of transport that is characterized by crammed mini-busses and vans (*matatus*) often for long distances making this form of mobility a perfect vector for the spread of respiratory diseases. But even after the government announced countermeasures to curb the spread of the disease, still slum residents are inadvertently affected.

Finally, our analysis establishes that social safety nets, when used in combination with changes in the above policies, have the potential of mitigating transmission of future pandemics. As per our model projection, lack of social security measures such as health insurance coverage can be exacerbated through societal inequalities and job insecurities given that most of the residents in slum areas rely on daily livelihoods without pension. Social control measures instituted under the strategy of containment included casual workers being subjected to compulsory leave days, yet there was no guarantee that one would be recalled back after the pandemic is over. For those who are into the private sector, the majority are absorbed into low-earning, high-risk jobs such as, waste recycling, street vending, and artisanship. For some, especially those residing in major cities of Nairobi, Kisumu, and Mombasa, the state’s brutality executed through police force meant that residents violate social control measures in order to circumvent the containment rules and earn a living. In view of this behavior, we observe that for future pandemics, the PHI strategy should be integrated with these patterns of livelihoods. The strategy should also entail a social sensitivity element such as gender, age, and social class.

These social considerations should be an integral part of the future response strategy to pandemics. Our observation concurs with JP Morgan’s (cited in Mail online 2020) findings that the enforcement of lockdown strategies did not necessarily lead to reduction in the rate of transmission of COVID-19. There seem to have been “other” pre-existing social, economic, and political conditions contributing to the spikes. Related to this “other” factor is the question of socio-economic injustice. The declaration by the Ministry of Education for all schools and colleges to shift to online learning as part of the containment strategy was yet another burden to the slum residents. Previous studies have clearly shown how the Kenyan education system perpetrates inequalities across the entire ecosystem—staff, facilities, and equipment (Alwy and Schech 2007). It should, however, be noted that, although previous studies show strong correlation between closure of schools and workplace and significant reduction in the transmission of influenza (Koo et al. 2020), our model projection points to the contrary; enforcement of such draconian strategies could actually trigger structural inequalities. In general, our model suggests that any PHI strategy which excludes social geometry either directly or indirectly is bound to be rejected and is also subject to inequity. This failure in strategy is exacerbated by the design of the current PHIs. What is perhaps missing from the current PHI strategy is a context-specific structure that is more explicit on the resident’s social geometry building blocks—location, distance, and direction.

### Simulating Decision in Selecting the Most Effective PHI Strategy

This paper also explains how the variables (henceforth referred to as ‘risk factors’) in Annex 5 would be affected by various PHI strategies (containment, lockdown, and social pendulum) on pairwise ranking technique. The severity of the ‘risk factors’ is based on the discourse analysis as outlined by the *Indicator Development for the Surveillance of Urban Emergencies* (IDSUE) classification (USAID/Concern World Wide 2014). In our modeling, the ranking would then facilitate decision making in selecting the most effective strategy in managing future pandemics. On this technique, the most effective strategy is one with the highest frequency of the ‘Green’ code, while the ‘Red’ would symbolize inappropriate or potentially harmful strategy. To be precise, for the three strategy options, the 16 items were compared in the decision matrix (Annex 5), such that the rankings generated the prescribed strategy option.

The number of times a ‘risk factor’ had been found to be most affected by a particular ‘reagent’ (strategy option) was determined by counting the number of times a distinct color appeared in the decision matrix (“Red,” “Yellow,” or “Green”). Each one of the ‘reagents’ was mutually exclusive. The assumption here is that the ‘reagent’ would be introduced at different times to the same group of people. Residents’ reaction would vary according to the reagent’s effect. The outcome of the decision matrix (Annex 5) facilitated the construction of Table 1, with each ‘risk factor’ being compared against the three strategy options. Thus, ‘containment’ was compared first with ‘lockdown.’ We deduced that ‘containment’ induced the least (1 out of the possible 16), ‘high risk factor’ compared to ‘lockdown,’ which generated the highest (13 out of possible 16) ‘high risk’ factors followed by ‘social pendulum’ with three ‘high risk’ factors. In line with our model projection, if movement restrictive strategies, such as lockdown and containment, are instituted, most of the items would indicate ‘high risk’ and ‘medium risk,’ respectively. However, urban infrastructure and slum collectivism seem not to trigger ‘high risk’ on the same strategy. Interestingly, ‘infrastructure,’ ‘collectivism,’ and ‘asymptomatic’ factors would actually change to “high risk” if the *social pendulum* was to be adopted as the strategy option for managing COVID-19 and other unforeseeable pandemics. The strategy option recording the highest number of risk factors, is considered to be the least preferred option. In this case, ‘lockdown’ appears to record the highest (13) in the decision matrix than any other strategy option (Table 1). Hence, the public health officials and government authorities would be advised to be cautious of a ‘lockdown’ as a PHI strategy option.

**Table 1:** Results of simulating decisions for the PHI strategy options

Policy option	High risk	Medium risk	Low risk	Score	Rank
Containment	1	15	0	1	3
Lockdown	13	0	3	13	1
Social Pendulum	3	1	12	3	2

*Source* Table 1 is a derivative of Annex 5, simulating the three strategic interventions against the 16 items (‘risk factors’) in the Nairobi informal settlement

In line with our model projection, ‘lockdown’ strategy option was considered to be the most problematic. From Table 1, it is understood that although the *social pendulum* option generated the highest number (12 out of 16) of ‘low risk’ factors, its adoption would have to consider three structural conditions:

- if it is adopted as a *solitary* PHI strategy, it is likely to put pressure on existing outdoor infrastructure (watering points, roads, and other public amenities), unfortunately, leading to outdoor pollution;
- the epidemiological management of the asymptomatic condition among the residents will be crucial. As illustrated in the decision matrix (Annex 5), this ‘risk factor’ is likely to be highest for both ‘containment’ and ‘lockdown’ strategies; and
- Although, the asymptomatic factor, would indicate ‘medium risk’ for *social pendulum strategy,* this model outcome points to the difficulties that are likely to be encountered by public health officials in the surveillance and detection of “hidden” cases in densely populated areas.

In the event that all the three strategy options fail to curb community transmission through asymptomatic individuals, a combination of interventions should be integrated in the PHI strategy, including surveillance, school closure, and workplace spatial distancing. Our modeling outcome here concurs with a recent study by Qun and his colleagues, who recommended that in situation of a persistent asymptomatic conditions, potential secondary control-response strategies should be part of the intervention (Qun et al. 2020). In line with our model projection, the prevalence of future pandemics will be mainly driven by the resident’s access or lack of access to a combination of both public health facilities (sanitation and air quality), socio-economic safety nets, and appropriate urban planning that accommodates the unique behavioral patterns of people living in informal settlements.

Given the conditions under which slum residents live we consider what ‘containment’ means for the vulnerable population in the informal settlements. In order to look more systematically at the question of a pandemic management in informal settlement, a new concept is introduced here, that of ‘social pendulum.’ The idea of ‘social pendulum’ is based on both the principles of physics (time, space, location, distance, and direction) and social science principles (relationships, social space, solidarity, and shareability). The *swing* symbolizes the residents’ coping strategies. The ‘pendulumic’ analogy we propose in this paper forms part of the alternative PHI strategy developed in the following section.

### Social Pendulum: An Alternative Strategy?

The qualitative modeling of an alternative framework in the foregoing section indicates that such constructs can shed some light on the human behavior and what type of PHI strategy would be ideal for the urban informal settlements.

The ‘urbanites’, and especially the slum residents’ (henceforth referred here as *slumites*) behavior and cultural values strongly influence the acceptability of any PHI strategy. For instance, individuals or groups who are *socially close* to each other will handle the pandemic differently than those who are socially distant. The PHI strategy proponents may decide to follow this pattern of behavior or simply impose a generic scheme designed without considering the contextual dynamics. This decision determines the outcome of a strategy. In this paper, we conceptualize the actions and behaviors of both the *slumites* and the public health officials as changes in social geometry. In this light, ‘containment’ strategy that was instituted by the Kenyan government to prevent transmission of COVID19 is viewed as a *conflict* that is caused by disrupting social relations. In the social space parlance, such changes are labelled deviant interventions because they alter the social geometry balance of power in a community. On this account, it is plausible to argue that ‘containment’ strategy disrupted the structure of *slumites* in different directions, location, and distance. As observed by Black (2011, pp. 6), “the severity of this disruption is a direct function of the magnitude of the change.” On the basis of this explanation, it is reasonable to observe that the containment strategy gave rise to different forms of social geometries. It is therefore necessary to analyze the importance of these socio-geometric (im)balances with the aim of *reimagining* an effective PHI strategy in the management of future pandemics. So, what should constitute an effective PHI strategy in urban slums?

To address this question, our study suggests that an effective PHI should aim at (1) closing the *swing loop*—in short, provide the needed basic requirements to the residents, including watering point, movement corridors, sanitizers, and indoor ventilation facilities; and (2) intervene *just-in-time and space* (JITS). JITS approach to the management of public affairs, including pandemic would aim at minimizing overcrowding by ensuring that the PHI is provided wherever the immigrants are found along the *swing* pathway. However, such a strategy should be mindful that intervening on the basis of JITS may not necessarily be the panacea for preventing the transmission of a pandemic, because this is a logistic intensive process that depends on the efficiency of the existing public health infrastructure, road connectivity index, and quality of housing. In the world of public policy, lack of these facilities may actually lead to spikes in transmission of an infectious disease, leading to a potential humanitarian disaster. Previous studies that have examined other control measure models beyond the draconian ones (containment and lockdown) recommend effective monitoring and surveillance capabilities (Ng et al. 2020).

Yet, models that promote solidarity among people within same socio-economic stratum such as cooperatives have been found to promote inclusion (Borda-Rodriguez and Johnson 2020). In the current study, we utilize Black’s (2011), four socio-geometric techniques (form, style, quantity, and multidimensionality of social relations), to re-imagine an effective PHI strategy for managing pandemics. Form—the analysis and the design of an effective PHI strategy should allow the stakeholders to see how the relationship fits together and how the intervention serves the intended function of preventing transmission of the pandemic (COVID-19). Style—ideally, the explanation and the design of an effective PHI strategy should be able to account for the unintended consequences of the intervention. An important consideration is that when containment of collective communities in the informal settlement is intensified, hence, increasing the flow of people *indoors* (see Annex 3a), the risk of transmission goes high. To avert this, the PHI system should have the capability to trigger *outward swings* (see Annex 3b), which eventually increase social closeness of the *slumites*. This system thinking approach concurs with our model prediction that the closer the social distance, the more likely the homogenous group will collaborate with public health officials in instituting the PHI. As earlier illustrated in our modeling outcome (Annex 3b), spatial distance has minimal influence on survival of slum communities, because they can still retain connections through social informal networks.

Quantity—One of the principles of ethnomethodology is that the interpretation of the intervention should be bestowed upon the size of the affected population. Related to quantity is the last technique—multidimensionality of relationship—an understanding of the urban socio-cultural structures and their functions in the community helps the interventionists to make sense of people’s preferred approach to solving their problems. Therefore, in analyzing and designing an appropriate PHI strategy, caution must be made to ensure no new problems are created. Similarly, the egalitarian social organization, common among the slum communities can pose a challenge in managing intergroup dynamics. To make sense of the intergroup dynamics, Black (2004) analytical framework provides five elements that we think are useful in our new proposed social pendulum framework: (1) interventions should ensure high intimacy and interdependence between members; (2) the intervention should not alter social geometries, groups should maintain the social closeness; (3) the intervention should be functionally interdependent; (4) create or sustain cultural closeness among the population; and (5) groups can be separated by an intermediate degree of relational distance.

The above criterion is key in ensuring that the group configuration is intact and that the public health interventionists build up on the existing social geometries. However, with the complex nature of urban slums, it is not feasible to have a ‘one-fit-all’ approach to PHI. In the foregoing discussion, the profile of *slumites* reveals that their pattern of livelihood is *erratic* (unpredictable), and their coping mechanisms and livelihood activities are *multi-directional* (see Annex 3a, b). As a result, the residents can hardly follow a systematic order of events. Rather, their lifestyle maintains constant *swings.* Worse still, the swings (movement) are not linear, but the day-to-day needs pushe them to *swing* between their *makeshift* houses and the “unknown” destinations, and back. In this paper, the *swings* represent the socio-economic needs of the residents, while the *makeshift* houses represent the fixed points akin to the massless rod of the physical pendulum. Hence, we coin the notion ‘social pendulum,’ figuratively to represent the PHI *structure* that is anchored on realities of the target population. In our conception of the new framework, the *swings* perform three major functions in the new configuration of the slum ecosystem:

(i) The idea of ‘social pendulum’ allows *slumites* to ‘swing’ freely depending on the time, available, spatial space, location, distance, direction, and access to livelihood opportunities;
(ii) The swings symbolize the residents’ coping strategies against socio-economic shocks and health risks associated with high concentration of people within a limited spatial space; and
(iii) The swings allow the community to maintain social networks and identity.

**Fig. 6:**
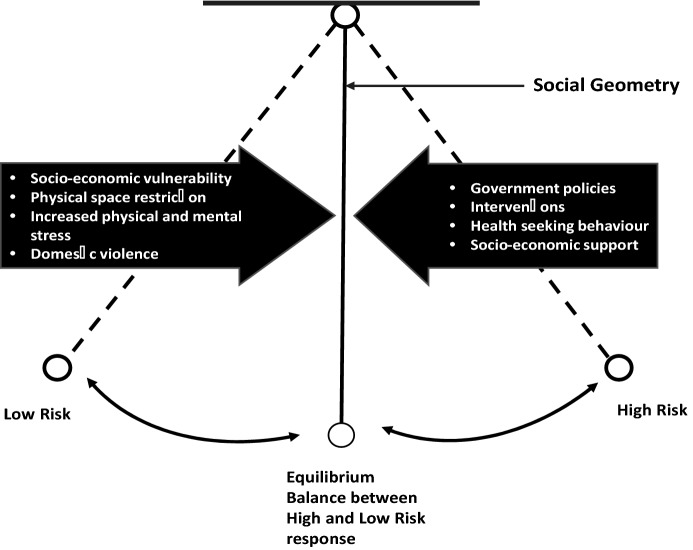
A New Framework (*Social Pendulum*) for Informing PHI Strategy in Urban Slums

From this characterization, it is plausible to observe that an effective intervention should then be one that allows diffusion of the population away from the ‘community nuclei.’ Essentially, our proposed model offers a canal pathway for decongesting the spatial space, at the same time sustaining *social closeness* of the *slumites* for their socio-economic survival. This ‘pendulum-like’ movement (see Fig. 6) of people as they seek livelihood opportunities has the potential of creating indoor spatial space, which in turn improves indoor ventilation. This approach to the management of pandemics in urban slums can be helpful in lessening the ecological exigency on sanitary facilities. These socio-environmental conditions are known to prevent spread of respiratory illness (Dianati et al. 2019). Figure 6 summarizes the new proposed analytical framework for informing PHI strategy.

## Conclusion

In this paper, we have attempted to develop a theoretical framework which can be utilized to inform the development of a public health intervention (PHI) in urban slums of developing countries. We believe that gaps in the PHI not directly addressed by the new proposed paradigm can also be addressed by social geometry. But neither the social geometry nor our newly proposed framework (social pendulum) is a theory. What the social pendulum framework enables us to do is to shed new insight on ideas drawn from social geometry, taking into account the minimalistic nature of epidemiological theories that are not broad enough to address public health issues in a rapidly changing urban environment. Our recommendation for adopting this model is in line with development institutionalists’ perspective, who caution that the success of any development model depends not only on the characteristics of the design, but also the adaptability to the context and should be socially embedded through the process of institutional bricolage (Rusca et al. 2015). Therefore, the scenarios developed in this paper and the proposed analytical framework are not presented as the absolute model for reference by public health interventionists. In any case the burden of disease in less developed countries is too complicated to be treated by a simple framework like the one we develop in this paper. To our knowledge, there are currently no programs or research focusing on the social changes in regard to mobility and livelihood taking place in slum areas from a public health point of view. This lack of attention presents a pressing conundrum and potentially viable opportunity for further research.

## Figures and Tables

**Annex 2:**
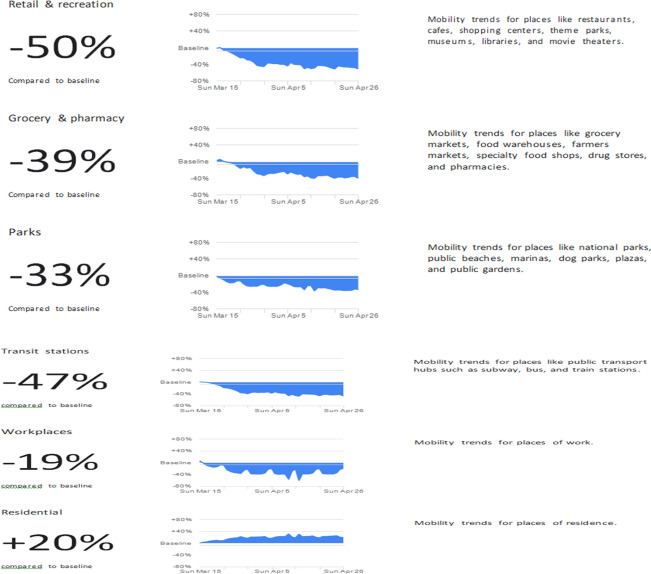
Google mobility data as of 26 April 2020. *Source:* Google Mobility Data April 26, 2020. Available at: https://www.google.com/covid19/mobility/. Accessed September 30, 2020

**Annex 3:**
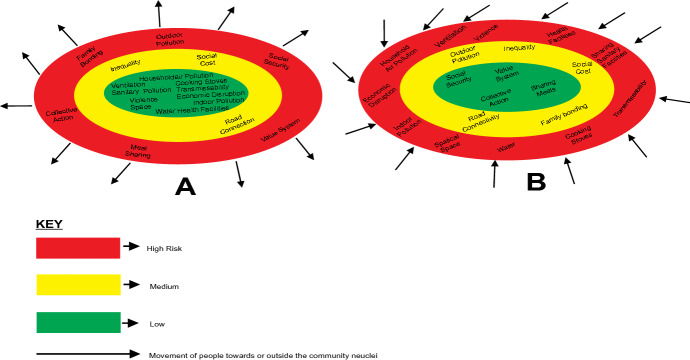
**a**, **b** Simulating behavior of slum residents before (**a**) and after (**b**) the introduction of the containment strategy

**Annex 5:**
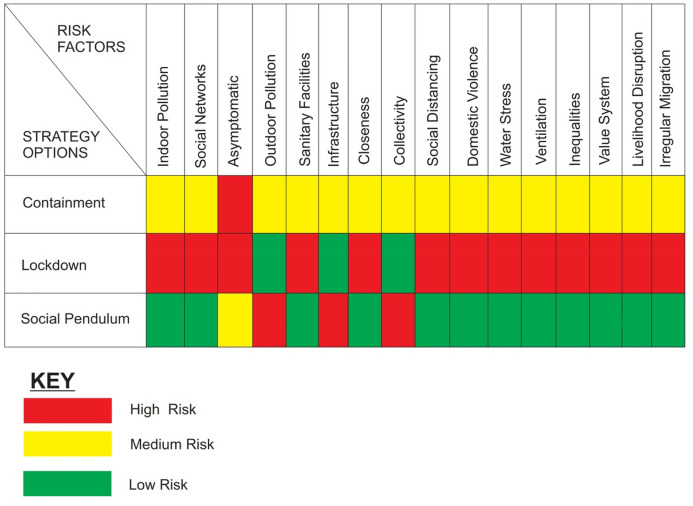
Simulating decisions on the basis of the three strategic interventions against the 16 items (“risk factors”) in the informal settlement, Nairobi Kenya

## Annex

See Annexes 1, 2, 3, 4, and 5.

**Annex 1:** Covid-19 cases in Kenya from March 13 to May 28, 2020

Date	Daily cases	Increase in daily new cases (%)	Sum of cases	Date	Daily cases	Increase in daily new cases (%)	Sum of cases
March 13th	1		1	April 20th	11	37.5	281
March 15th	2	100	3	April 21st	15	36.4	296
March 17th	1	− 50	4	April 22nd	7	− 53.3	303
March 18th	3	200	7	April 23rd	17	142.9	320
March 19th	0	− 100	7	April 24th	16	− 5.9	336
March 20th	0	0	7	April 25th	7	− 56.25	343
March 22nd	8	Undefined	15	April 26th	12	71.4	355
March 23rd	1	− 87.5	16	April 27th	8	− 33.3	363
March 24th	9	800	25	April 28th	11	37.5	374
March 25th	3	− 66.7	28	April 29th	10	− 9.1	384
March 26th	3	0	31	April 30th	12	20	396
March 28th	7	133.3	38	May 1st	15	25	411
March 29th	4	− 42.7	42	May 2nd	24	60	435
March 30th	8	100	50	May 3rd	30	25	465
March 31st	9	12.5	59	May 4th	25	− 16.7	490
April 1st	22	144.4	81	May 5th	45	80	535
April 2nd	29	31.8	110	May 7th	25	− 44.4	607
April 3rd	12	− 58.6	122	May 8th	14	− 44	621
April 4th	4	− 66.7	126	May 9th	28	100	649
April 5th	16	300	142	May 10th	23	− 17.9	672
April 6th	16	0	158	May 11th	28	21.7	700
April 7th	14	− 12.5	172	May 12th	15	− 46.4	715
April 8th	7	− 50	179	May 15th	23	53.3	781
April 9th	5	− 28.6	184	May 18th	25	8.7	912
April 10th	5	0	189	May 20th	66	164	1029
April 11th	2	− 60	191	May 22nd	52	− 21.2	1161
April 12th	6	200	197	May 23rd	31	− 40.4	1192
April 13th	11	83.3	208	May 24th	22	− 29	1214
April 14th	8	− 27.3	216	May 25th	72	227.3	1286
April 15th	9	12.5	225	May 26th	62	− 13.9	1348
April 16&17th	21	133.3	246	May 27th	123	98.4	1471
April 18th	16	− 23.8	262	May 28th	147	19.5	1618
April 19th	8	− 50	270				

*Source* Compilation from the Republic of Kenya, Ministry of Health Daily COVID-19 Reported Cases covering the period March 13 to May 28, 2020

**Annex 4:** Comparing population density in Nairobi sub-counties

Sub-county	Total population	Land area Sq. Km	Population density
Dagoretti	434,208	29	14,908
Embakasi	988,808	86	11,460
Kamukunji	268,276	11	25,455
Kasarani	780,656	86	9063
Kibra	185,777	12	15,311
Lang’ata	197,489	217	911
Makadara	189,536	12	16,150
Mathare	206,564	3	68,940
Njiru	626,482	130	4821
Starehe	210,423	21	10,205
Westlands	308,854	98	3167

*Source* Kenya National Bureau of Statistics-Population Census Result of 2019 & Kenya Demographic and Household Survey (KDHS)
